# Superior cervical gangliectomy exacerbates the aortic dissection in mice

**DOI:** 10.1371/journal.pone.0357281

**Published:** 2026-09-01

**Authors:** Zhonghui Zhu, Hao Jin, Jie Li, Li Yu, Meifang Cao, Tao Jiang, Yi Jin, Baohong Jiang

**Affiliations:** 1 Shanghai Institute of Materia Medica, Chinese Academy of Sciences, Shanghai, China; 2 School of Pharmacy, University of Chinese Academy of Sciences, Beijing, China; 3 School of Chinese Materia Medica, Nanjing University of Chinese Medicine, Nanjing, China; 4 Shenzhen Institute for Drug Control (Shenzhen Testing Center of Medical Devices), Shenzhen, China; Army Medical University, CHINA

## Abstract

**Background:**

Sympathetic innervation is fundamentally required for physiological aortic homeostasis. Though it plays a vital important role in aortic function, its specific role in aortic dissection (AD) remains unclear.

**Methods and results:**

In this study, we explored the role of the sympathetic nervous system in AD by examining the effects of superior cervical ganglionectomy (SCGx) in mice. Using β-aminopropionitrile (BAPN) induced AD mice model at pre-lesional stage, the tyrosine hydroxylase (TH) expression, indicative of sympathetic activity, increased early in AD progression and correlated with aortic elastic lamina degradation. SCGx did not affect aortic structure within 2 weeks without BAPN treatment, but bilateral SCGx exacerbated AD severity, increasing rupture rates and tear range after BAPN and angiotensin II (AngII) treatment.

**Conclusions:**

These findings highlighted the role of sympathetic nervous system in early AD stages and suggested bilateral SCGx may negatively impact AD progression. Furthermore, we found a trend toward smooth muscle cell phenotypic transformation after implementing SCGx in AD, revealing the integrity of the SCG played a pivotal role in preserving aortic homeostasis.

## Introduction

Aortic dissection (AD) is a life-threatening acute arterial disease with high mortality [1]. Our previous studies revealed that spatiotemporal differences in inflammatory cell infiltration during AD progression and targeted therapy for inflammatory cells offers a promising approach for AD treatment [2,3]. However, since infiltration of inflammatory cells and aortic rupture occur almost simultaneously, identifying earlier triggers of aortic rupture is crucial. Hypertension, a major risk factor for AD, is linked to sympathetic hyperactivity [4–7]. While, the role of sympathetic activity in AD development remains unclear.

The rhythmic activity of the heart is governed by the sympathetic nervous system, and specialized neural structures known as baroreceptor are present in the aortic arch, regulating the heart rate and blood pressure [8–11]. Thus, an inseparable relationship exists between the sympathetic nervous system and the cardiovascular system. It was found that clinical patients with AD exhibit abnormal levels of noradrenaline in the blood and a significant increase in systemic sympathetic nerve activity [12]. The timeline of sympathetic nerve activity in relation to AD onset remains unclear. Because modern medicine still lacks the ability to accurately predict the occurrence of AD [13], it is not known whether the changes of sympathetic nerve activity occur before AD or not. Briefly, whether sympathetic nerves are merely correlative or truly causal of AD requires further investigation.

The superior cervical ganglion is reported to be a part of cardiac plexus, which innervates the aorta [14]. Resection of the ganglion to block the nerves in the innervated area is an effective approach. Superior cervical ganglionectomy (SCGx) has been reported to alleviate myocardial infarction injury, including inflammation, cardiomyocyte hypertrophy, and global cardiac dysfunction [15]. Besides, SCGx attenuated vascular remodeling in spontaneously hypertensive rats and enhanced smooth muscle cell function [16], indicating a direct effect on blood vessels’ mechanical strength and structure, which may relate with further inflammation infiltration [17].

To identify early trigger of AD, we focused on the effect of local sympathetic denervation with SCGx on AD, investigating the pathological changes of the tyrosine hydroxylase (TH) during pre-lesional stage of AD and the important role of SCG on preserving aortic homeostasis.

## Methods

### Animals

All C57BL/6J were purchased from Beijing HFK Bioscience CO.LTD and bred in the animal care facility at Shanghai Institute of Materia Medica under a 12-hr light/dark schedule. All animal procedures were approved by the Institutional Animal Care and Use Committee (IACUC) of Shanghai Institute of Materia Medica (IACUC number: 2024-01-GDA-116 and 2023-10-GDA-105). Animal studies also followed the Guide for the Care and Use of Laboratory Animals published by the U.S. National Institutes of Health (NIH).

### AD mouse models

**Fig 1:**
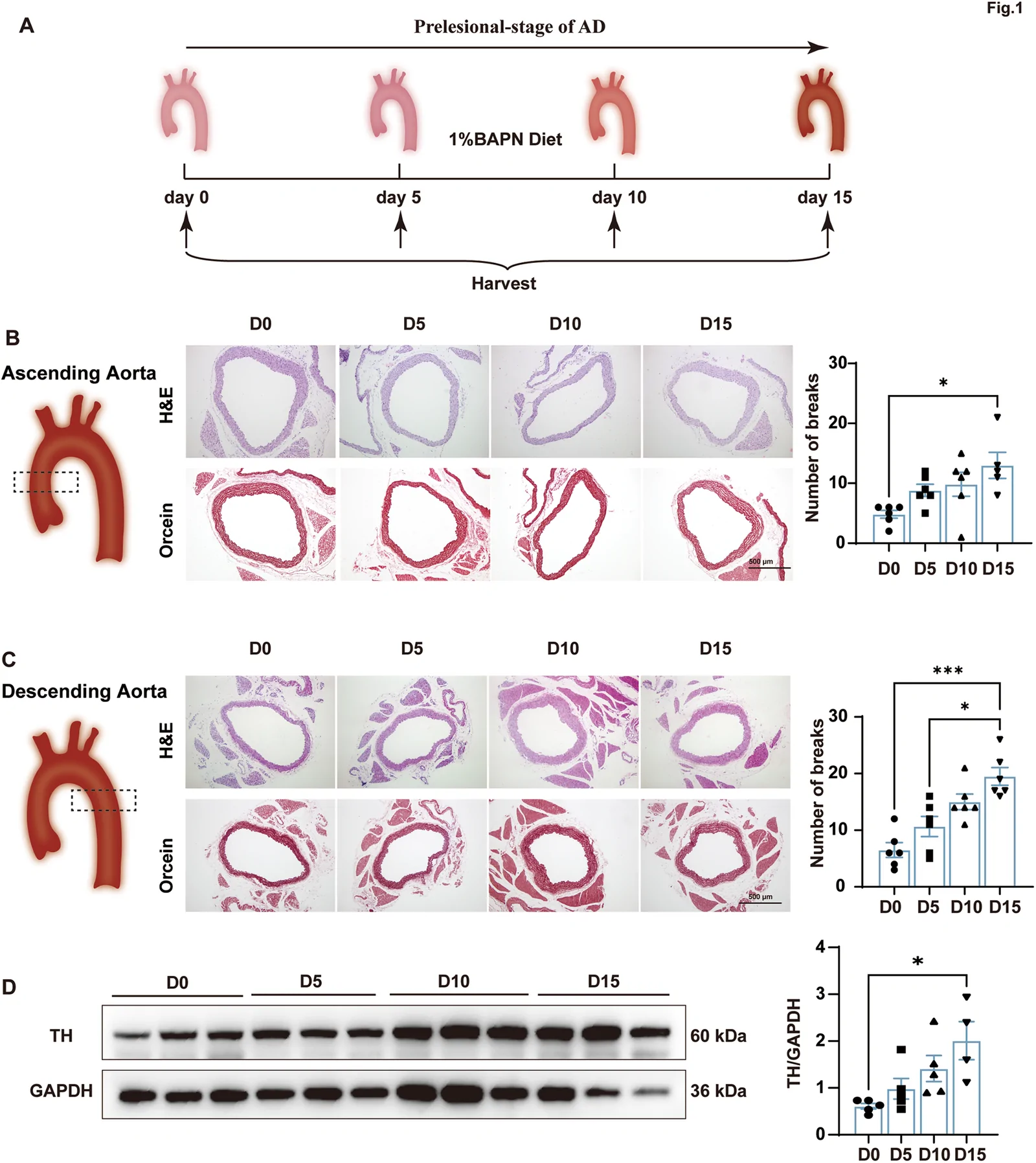
The TH levels exhibited significant upregulation during the pre-lesional stage of AD. (A) Experimental design. (B) Representative images of H&E and orcein stained on the ascending aorta and the quantification of the number of elastic laminae breaks (D0, n = 6; D5, n = 6; D10, n = 6; D15, n = 5). (C) Representative images of H&E and orcein stained on the descending aorta and the quantification of the number of elastic laminae breaks (n = 6/group). (D) Western blot analysis showing increased TH expression in the whole aorta (D0, n = 5; D5, n = 5; D10, n = 5; D15, n = 4). Data are presented as mean ± SEM with individual datapoints. Scale bars are shown on the images. Group differences were assessed using Kruskal-Wallis tests. * p < 0.05, ** p < 0.01, *** p < 0.001.

To evaluate the TH amount changes accompanied with AD progression at the pre-lesional stage [3], three-week-old male C57BL/6 mice were fed with formulated diet containing 1% BAPN (cat. A0796, TCI), aortas were sampled according to the duration of 1% BAPN diet (Fig 1A). And to evaluate the effect of SCGx on AD, the mice were fed with 1% BAPN diet after bilateral SCGx for the following 15 days, For the SCGx surgery, mice were anesthetized via intraperitoneal injection of xylazine hydrochloride (10 mg/kg, MeilunBio®) and tiletamine/zolazepam (50 mg/kg, Zoletil®50). To minimize suffering, daily health monitoring was performed to assess the need for post-operative analgesia. We specifically monitored for signs of distress, including ruffled fur, hunched posture, and decreased activity. All mice exhibited normal behavior and recovery after the operation, thus no additional analgesic intervention was required. 1.5 mg/kg AngII (cat. HY-13948, MedChemExpress) was administered subcutaneously at day 14, and mice in CON group were fed with a normal diet and injected with saline at the indicated time point for AngII (Fig 3A). At the endpoint of each experiment, mice were deeply anaesthetized with xylazine hydrochloride (MeilunBio®, 10 mg/kg) and tiletamine/zolazepam (Zoletil®50, 50 mg/kg).Once the absence of pedal withdrawal reflex was confirmed, mice were humanely euthanized by cardiac exsanguination, and the death was confirmed through absence of a heartbeat. After perfusing with saline through the heart, the entire aortas were separated for further analysis. The aortic dissection was confirmed by two steps. Firstly, for the obvious tear of the whole aorta, AD was evaluated under the stereomicroscope (SXZ7, Olympus Corporation, Japan). Secondly, to observe the slight tear, the ascending aorta was sliced and stained by haematoxylin–eosin (H&E). AD was characterized by the formation of false lumen or tear with the blood in the medial layer. The incidence of AD in every group was the combination of the two-step evaluation.

### Superior cervical ganglionectomy (SCGx)

All mice were anesthetized with an intraperitoneal injection of mixture of zolazepam/tiletamine (Zoletil^®^50, 50 mg/kg) and xylazine (10 mg/kg). A 2 cm incision was made along the midline of the neck. Using a dissecting microscope (SZX7, Olympus), the subcutaneous tissue and fat were separated to expose the bifurcation of the carotid artery. The superior cervical ganglion, identified as a white elliptical structure behind the bifurcation, was then dissected. Afterward, penicillin-streptomycin solution was applied to the wound to prevent infection. For the sham surgery, the ganglion beneath the bifurcation was identified and gently touched twice. Successful resection was indicated by blepharoptosis on the resected side of the mice.

### Western blot Analysis

The aortic tissues were lysed using a mixture of RIPA lysate and PMSF. A 30-µg protein sample was electrophoresed on an SDS-PAGE gel and transferred to a PVDF membrane (IPVH00010, Millipore). The membrane was then blocked with 5% skimmed milk and incubated overnight with primary antibodies against TH (1:2000, cat. sc-25269, Santa Cruz). The membrane was subsequently incubated with horseradish peroxidase-conjugated (HRP) anti-mouse secondary antibodies. Protein bands were detected with an enhanced chemiluminescence (ECL) kit (abs921, Absin) and quantified by ImageJ software.

### Aortic tensile strength

Detection of mechanical tensile strength of the aorta was performed according to our previous report^13^. Briefly, aorta was removed and washed with ice-cold saline. After trimming of perivascular tissues, the aorta as the indicated segment was sectioned transversely with about 1-mm length. The aortic rings were immersed in calcium-free saline at room temperature, then mounted and connected to a force transducer (Panlab S.L., Cornella, Spain). The tension required to rupture the aortic ring was collected using Animal Bio Amp and PowerLab 8/30 instruments data acquisition system.

### Histology and immunohistochemistry

The ascending and descending aorta was sectioned into 4 μm thick slices. The sections were subsequently stained with H&E or orcein or Sirius Red staining. For immunohistochemistry and Immunofluorescence, aorta sections were incubated with antibodies against TH (1:200, cat. sc-25269, Santa Cruz), CD68 (1:200, cat. BA3638, Boster), CD45 (1:200, cat. A00555-4, Boster), NFH (1:1000, cat. R25134, Zenbio) at 4ºC overnight, then incubated with HRP-conjugated secondary antibody for an hour at 37 ºC. For immunofluorescence, aorta sections were incubated with antibodies against α-SMA (1:200, cat.BM0002, Boster) and OPN (1:200, cat.BM4208, Boster) at 4ºC overnight, and then incubated with CY3-conjugated (1:100, cat.BA1032, Boster) and FITC-conjugated (1:100, cat.BA1105, Boster) secondary antibody for an hour at 37 ºC. Images were captured using an Olympus BX51 microscope and immunohistochemistry sections were then scanned by C13239-01 NanoZoomer S210. The number of elastic lamina breaks were counted through whole aorta. The percentage of elastic area was measured as the ratio of orcein stained area and whole aorta. The positions of the maximum and minimum wall thicknesses were chosen and repeated three times. For cell density, the number of nuclear stained by hematoxylin was counted per filed and total five different fields per tissue section were randomly selected at 400 × magnification. Immunohistochemistry staining was quantified as the ratio of positive area per filed for total five different fields. Observers blinded to the experimental conditions quantified the images using ImageJ.

### Statistical analysis

Data was expressed as mean ± S.E.M. Statistical analyses were performed using non-parametric tests. Comparisons between two independent groups were conducted using the Mann-Whitney U test. For comparisons involving three or more groups, the Kruskal-Wallis test was applied, followed by Dunn’s post-hoc test for multiple comparisons. No data points were excluded as outliers.

## Results

### The significant upregulation of TH in pre-lesional stage of AD

To elucidate the sympathetic innervation before aortic rupture, BAPN, a lysyl oxidase (LOX) inhibitor, was employed to establish pre-lesional stage AD model (Fig 1A). Histological analysis through Hematoxylin and eosin (H&E) and orcein staining was conducted. Progressive degradation of the aortic elastic lamina during BAPN administration was analyzed based on orcein staining. Compared with the mice in group of D_0_, statistical significance was detected in group of D_15_ on ascending aorta (p < 0.05, Fig 1B) and descending aorta (p < 0.001, Fig 1C). To assess sympathetic nerve innervation, we monitored the expression of tyrosine hydroxylase (TH), which is the rate-limiting enzyme in catecholamine biosynthesis. Western blot quantification of the whole aorta showed a time-dependent upregulation of TH protein, which mirrored the increasing number of elastic lamina fractures in the aorta, revealing a temporal correlation between sympathetic innervation and elastic degeneration at pre-lesional stage of AD (Fig 1D).

### SCGx did not affect the structure of aorta

**Fig 2:**
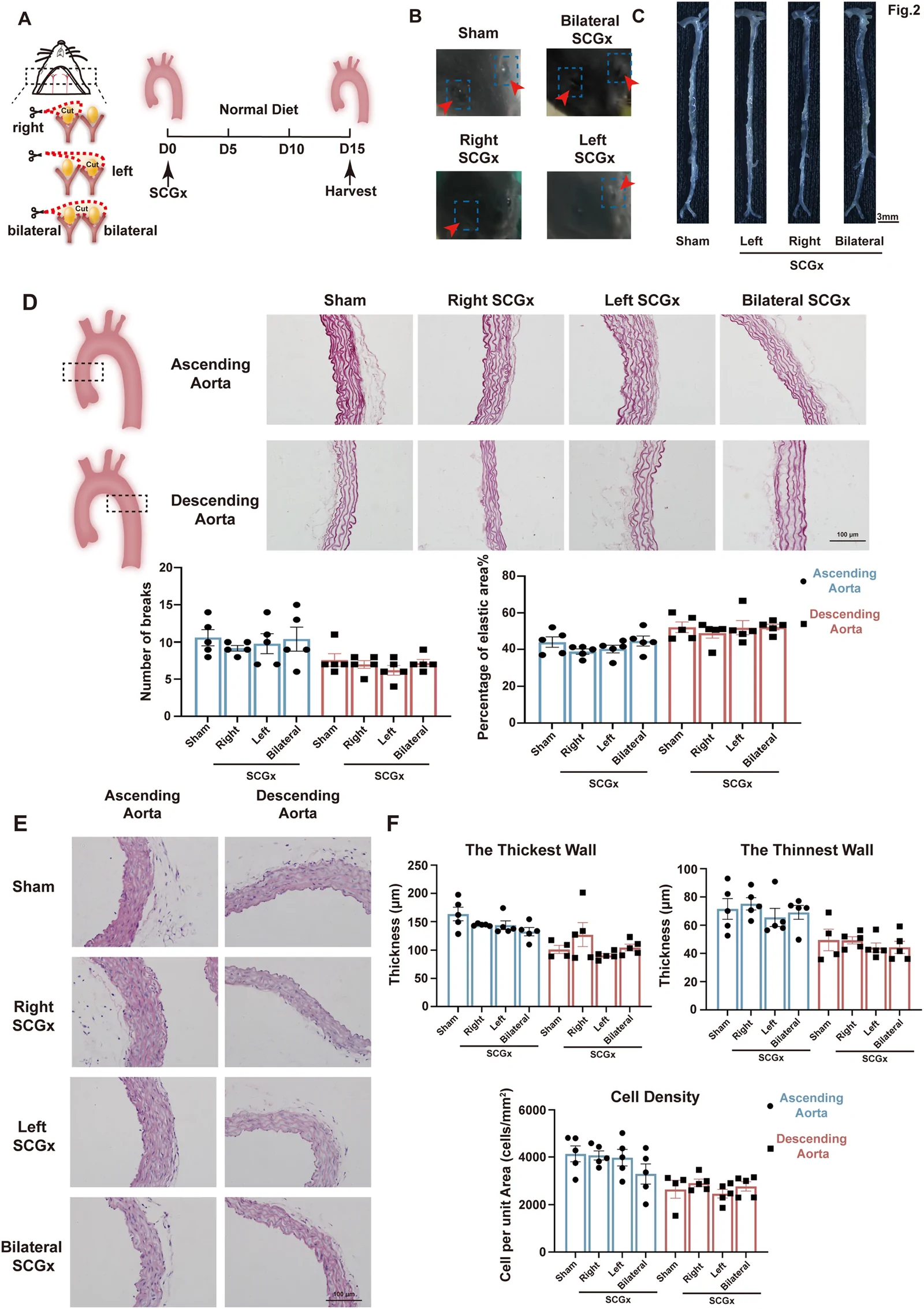
There was no impact of superior cervical ganglionectomy (SCGx) on the structure of the aorta. (A) Experimental scheme for the establishment of denervation model. (B) Images of blepharoptosis in each group of mice. (C) Representative images of aortas after SCGx operation. (D) Representative images of orcein stained ascending and descending aorta sections and the quantification of breaks and area of elastin lamina in each group of mice (n = 5/group). (E) Representative architecture of aortic wall (ascending and descending aorta) and (F) quantification of the thickest wall, thinnest wall and cell density. (n = 5/group). Data are presented as mean ± SEM with individual datapoints. Scale bars are shown on the images. Group differences were assessed using Kruskal-Wallis test.

Based on the finding of a time-dependent upregulation of TH protein during the pre-lesional stage of AD, SCGx surgeries were conducted to investigate the impact of sympathetic innervation on aortic structure (Fig 2A). Successful gangliectomy was confirmed by observing blepharoptosis. Surgery on each side caused drooping of the respective eyelid (Fig 2B). No mice died post-surgery, and no gross abnormalities were observed in the aorta after surgery (Fig 2C). Orcein staining confirmed that the arterial elastic lamina of both the ascending and descending aorta remained unaffected by SCGx (Fig 2D). Consistently, H&E staining also showed no significant structural alterations, such as in wall thickness or arterial cell density after surgery (Fig 2E-F).

### SCGx exacerbated the severity of AD

**Fig 3:**
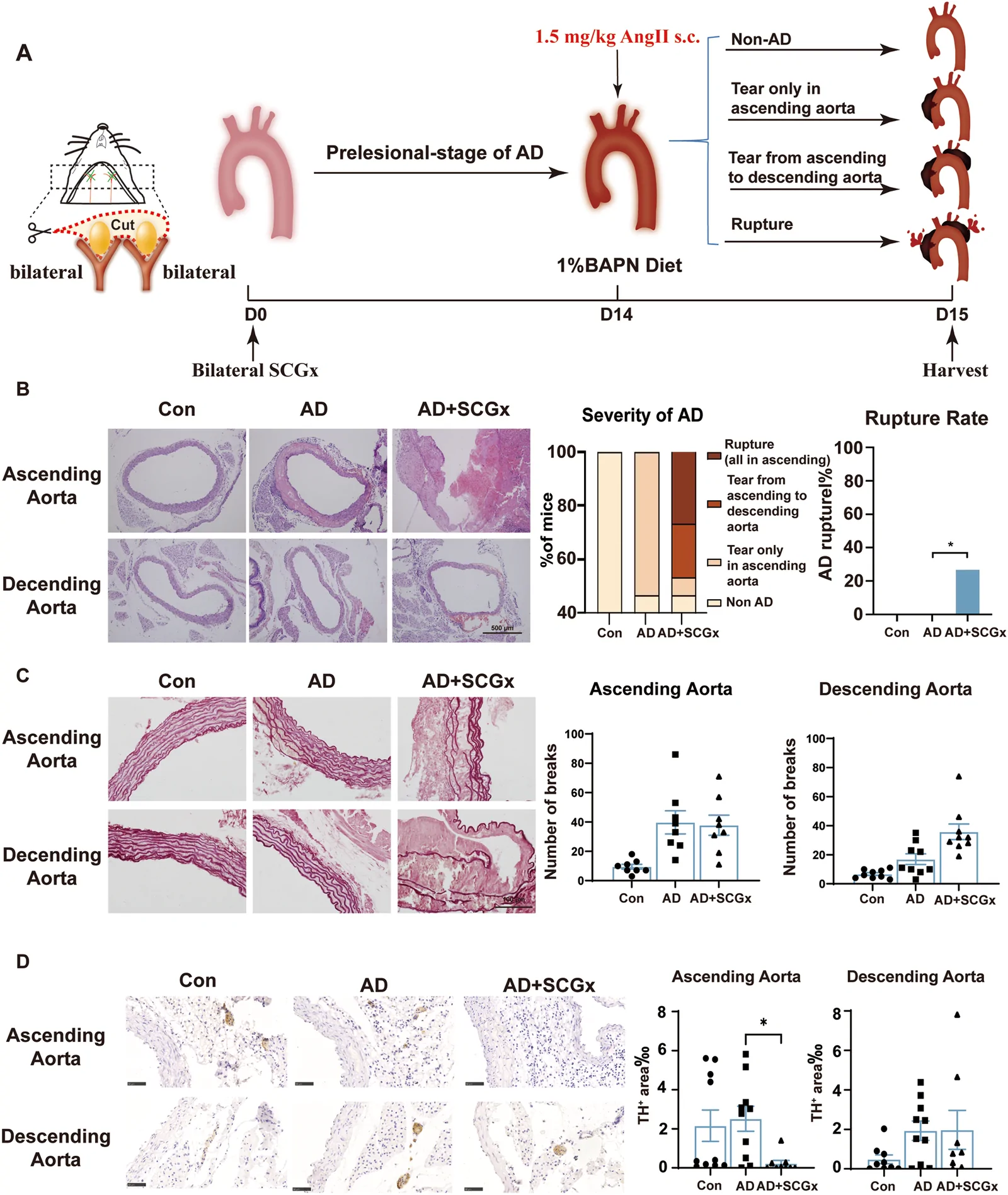
SCGx aggravated AD severity in mice. (A) Experimental scheme depicting the establishment of the animal model for AD and AD + SCGx. (B) Representative H&E stained sections of ascending and descending aorta, and assessment of the severity of AD and rupture rate (n = 15/group). (C) Representative orcein stained sections of ascending and descending aorta and quantification of elastic laminae breaks (For ascending aorta, Con, n = 8; AD, n = 8; AD + SCGx, n = 8; For descending aorta, Con, n = 9; AD, n = 9; AD + SCGx, n = 8). (D) Representative images of the immunohistochemistry of TH and quantification of TH^+^ area in the ascending (n = 10/group) and descending aorta (Con, n = 10; AD, n = 10; AD + SCGx: n = 8). Both Con and AD group were performed sham surgery. Data are presented as mean ± SEM with individual datapoints. Scale bars are shown on the images. Group differences were assessed using one-way ANOVA for continuous variables and Kruskal-Wallis test for scaled variables. * p < 0.05, ** p < 0.01, *** p < 0.001.

Although the possible effects of SCGx on the aorta within 15 days were excluded, its potential impact on AD progression remains a critical question warranting further investigation. To explore this, mice underwent bilateral SCGx and were subsequently fed with 1% BAPN for 15 consecutive days. One day before sampling, AngII was administered, and aortic structural assessments were then performed (Fig 3A). H&E staining revealed that in the AD group, the tear was limited to the ascending aorta, whereas in the AD + SCGx group, the tear extended from the ascending aorta to the descending aorta, with a significantly elevated rupture rate (p < 0.05, Fig 3B). Orcein staining showed that the number of elastic laminae breaks in the ascending aorta were similar in the AD + SCGx group and the AD group. For the descending aorta, the number of elastic laminae breaks in the AD + SCGx group exhibited an increasing trend compared to the AD group (p = 0.093, Fig 3C). Immunohistochemical staining indicated a significant reduction in TH^+^ area in the ascending aorta of the AD + SCGx group compared to the AD group (p < 0.05). In the descending aorta, no significant differences between the Con group and the AD group were found. (Fig 3D). Further detection of the superior cervical ganglion showed a significant decrease in neurofilament heavy chain positive area in the AD group compared to the Con group (p < 0.05, Supplementary Fig S1 in S2 File).

**Fig 4:**
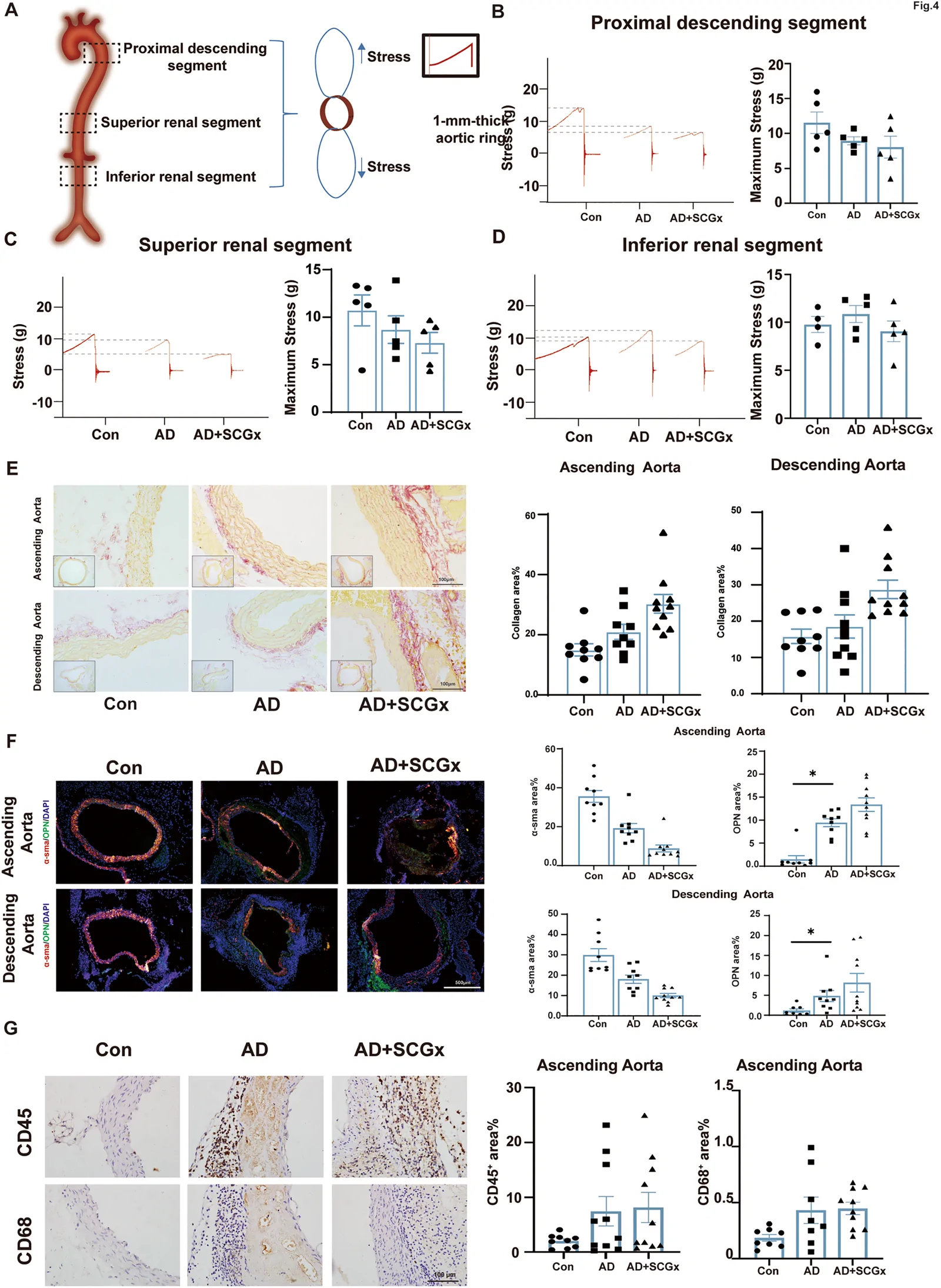
SCGx did not change the aortic tensile strength, collagen deposition, VSMC phenotypic transition and inflammation infiltration during AD. (A) Schematic diagram of aortic segments and method of aortic tensile strength. (B) Aortic tensile strength at the proximal descending segment (n = 5/group), (C) the superior renal segment (n = 5/group) and (D) the inferior renal segment (Con, n = 4; AD, n = 5; AD + SCGx, n = 5). (E) Representative images of Sirius Red staining on ascending aorta (Con, n = 9; AD, n = 9; AD + SCGx, n = 10) and descending aorta (Con, n = 9; AD, n = 10; AD + SCGx, n = 10) and its quantification of collagen area. (F) Multi-immunofluorescence staining of α-SMA and OPN on ascending aorta and descending aorta and its quantification of each proportion of area (Con, n = 9; AD, n = 9; AD + SCGx, n = 10). (G) Representative images showed CD45 and CD68 immunohistochemical staining, and quantification of the ratio of the CD45^+^ (Con, n = 9; AD, n = 10; AD + SCGx, n = 10) and CD68^+^ (Con, n = 9; AD, n = 9; AD + SCGx, n = 10) area in ascending aorta. Data are presented as mean ± SEM with individual datapoints. Scale bars are shown on the images. Group differences were assessed using Kruskal-Wallis test.

To investigate the mechanisms underlying the exacerbation of AD following SCGx, we evaluated aortic mechanical properties, extracellular matrix remodeling, smooth muscle cell phenotype, and inflammatory cell infiltration. The tensile strength of the proximal descending, superior renal, and inferior renal aortic segments did not differ significantly between the AD and AD + SCGx groups (Fig 4A-D). Likewise, Sirius Red staining showed comparable collagen deposition in both the ascending (p = 0.181) and descending aorta (p = 0.054) between the two groups (Fig 4E). Although the AD + SCGx group exhibited numerical changes in α-SMA and OPN expression relative to the AD group, none of these differences reached statistical significance in either the ascending (p = 0.086 for α-SMA) or descending aorta (p = 0.118 for α-SMA, Fig 4F). Similarly, no significant differences in CD45⁺ or CD68 ⁺ cell infiltration were detected either between the Con and AD groups or between the AD and AD + SCGx groups (Fig 4G). In addition, multi-segment H&E staining demonstrated that SCGx did not alter the total number of ganglia or nerve bundles (Supplementary Fig. S2 in S2 File). Collectively, despite aggravating AD severity, SCGx was not associated with statistically significant alterations in aortic tensile strength, collagen deposition, vascular smooth muscle cell phenotypic markers, or inflammatory cell infiltration.

## Discussion

**Fig 5:**
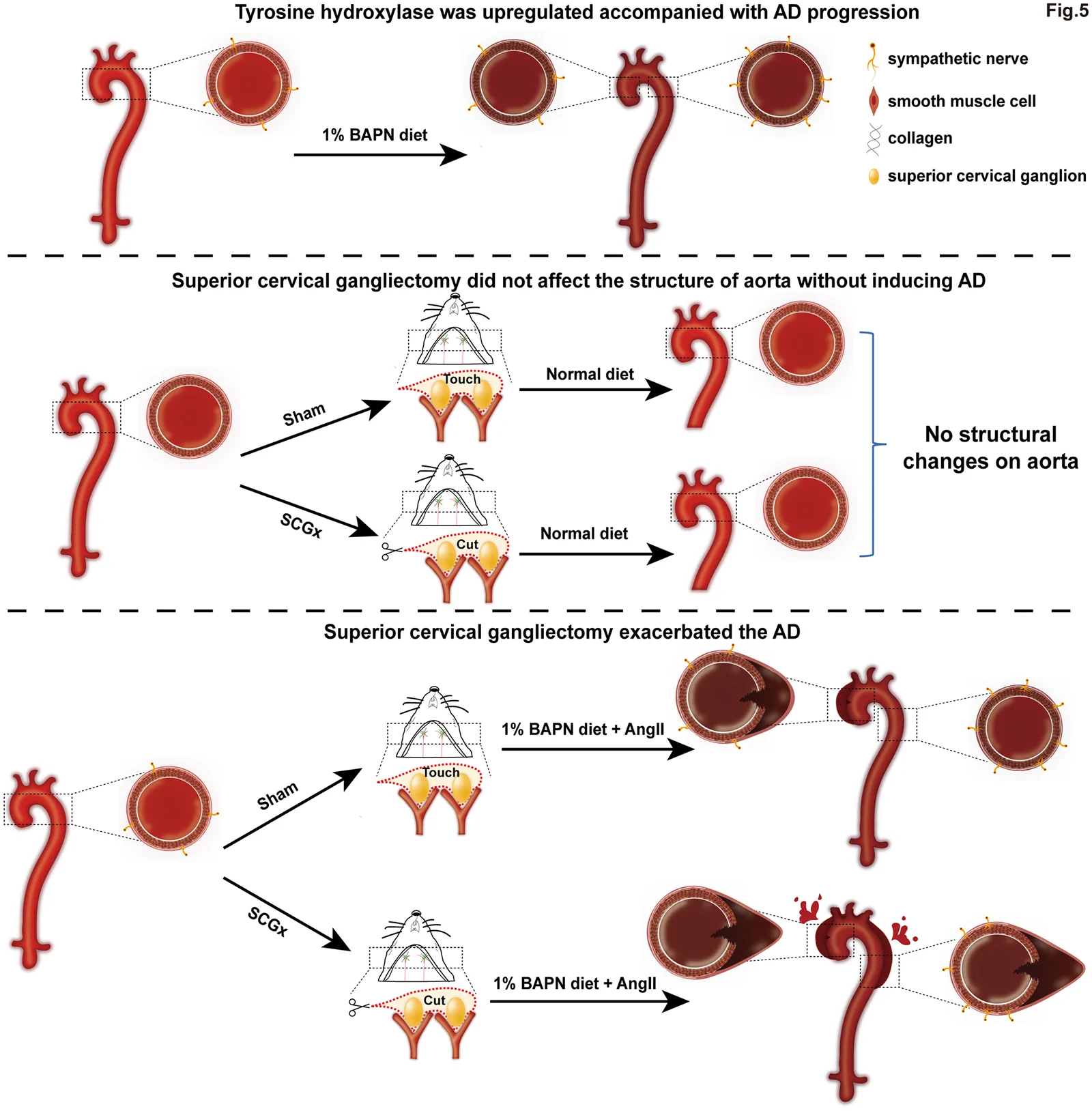
Graphical Abstract. This study demonstrates elevated TH expression during early AD and SCGx worsens AD rupture.

Our present study has unveiled two key insights into the pathophysiology of AD. First, we observed a significant increase in TH expression during the early stages of AD. Second, SCGx surgery, while not notably altering the aortic gross structure within 2 weeks, markedly worsened AD progression by increasing aortic rupture rates and enlarging tear range (Fig 5). These findings highlight the critical involvement of the sympathetic nervous system in the early pathogenesis of AD and imply that SCGx may adversely influence disease progression. However, the exact downstream molecular mechanisms by which SCGx exacerbates AD remain to be fully elucidated and are subject to further investigation. The observed TH upregulation might also represent a non-specific stress response to vascular injury.

Our results are in line with the findings of Kamra et al., SCGx did not affect chemoreflex responses to hypoxia and normoxic hypercapnia in either group without inducing acute lung injury model, but closely associated with chemoreflex sensitivity after modeling [18]. In the pre-lesional stage of AD, TH expression exhibited a significant upward trend, which was positively correlated with the fracture of the aortic elastic lamina. This finding holds potential significance as it underscores the role of the sympathetic nervous system in the pathophysiological process of AD. TH, as a rate-limiting enzyme in catecholamine biosynthesis, serves as a key marker of sympathetic nerve innervation and activity [19]. The observed increase in TH expression suggests that the sympathetic nervous system is activated in the early phase of AD. This overactivation may lead to a series of pathological changes, such as enhanced vascular smooth muscle cell contraction and disrupted extracellular matrix metabolism, thereby exacerbating the stress on the aortic wall and promoting the break of the elastic lamina [20,21]. However, whether this change contributes directly to disease progression, represents a compensatory response, or simply reflects a non-specific response to vascular injury remains to be determined.

Currently, there are three most common methods of neural intervention. The first method involves local nerve anesthesia, which temporarily blocks ganglia and may excessively interfere by blocking other nerves, potentially affecting the results of studies on specific ganglia [22]. The second method is systemic nerve removal by injecting compounds. A commonly used compound for sympathetic nerve removal is 6-hydroxydopamine (6-OHDA), which is taken up by dopamine transporters in neurons and specifically damages catecholaminergic neurons through an oxidative stress response [23]. 6-OHDA was first used to induce a Parkinson’s disease model [24]. However, research had found that the thoracic aorta had a low responsiveness to 6-OHDA [25]. The third method involves direct resection of the ganglion. SCGx had been specifically documented for more than seven decades [26]. SCG innervates a wide range of areas, including the pineal gland, pituitary gland, carotid body, thyroid and parathyroid glands, as well as the iris and eyelid [27]. Therefore, it is widely used in autonomic nerve research and is considered a practical denervation method. In our study, we adopted surgical resection to more accurately investigate the effect of SCG on AD.

Although SCGx did not exert a significant effect on the gross structure of the aorta within 2 weeks post-surgery, our study observed that it significantly intensified the severity of AD. This indicates that SCGx may influence certain intricate mechanisms, thereby exacerbating the pathological progression of AD. One possible explanation is that SCG surgery disrupts the normal regulatory function of the cardiac plexus on aorta, resulting in vascular tension imbalance. Wang et al. elucidated that the sympathetic nervous system regulates vascular tone by modulating the contraction and relaxation of vascular smooth muscle cells [28]. SCGx may compromise this delicate regulatory mechanism, causing the vessels to exist in a state of excessive contraction or relaxation. In addition, the different TH expression patterns observed between the ascending and descending aorta following SCGx underscore the major innervation of SCG on ascending aorta [14]. This neuroanatomical distinction may explain why AD extend to both ascending and descending aorta in AD + SCGx group. The compromised structural integrity of the denervated ascending aorta likely promotes more extensive tear propagation. In addition to vascular tension imbalance, SCG innervates a wide range of areas, including the pineal gland, pituitary gland, carotid body, thyroid and parathyroid glands, as well as the iris and eyelid [29], which may also contribution to the severity of aortic dissection.

Baroreceptors, a specialized neural structure, are located around the aortic arch. Like the carotid body, these baroreceptors sense changes in aortic blood pressure and transmit this information to the central nervous system, helping to regulate blood pressure [30,31]. Imaging studies have shown that the baroreceptor, which encircles between the left subclavian artery and the common carotid artery, detects blood pressure through specialized nerve endings and travels along the vagus nerve to the neck, ultimately reaching the nodose ganglion which extensively connected with SCG through cell bridge variants in mice [32–34]. When we performed SCGx, it may affect nodose ganglion, leading to nerve injury. This disruption could result in aortic wall remodeling and weakening of the aorta, potentially exacerbating AD [21]. Therefore, SCGx may not only impact the aorta directly but also indirectly influence the aorta through its effects on other organs and tissues. Our research findings reveal distinct contrasts with established literature in some aspects. Unlike the observations reported by Liu et al., SCGx exerted a markedly divergent effect on the pathophysiological trajectory of AD, highlighting the functional diversity of the same structures across species [35]. As an integral component of the autonomic nervous system, the SCG likely contributes to normal physiological functions and tissue homeostasis prior to AD onset. Consequently, resection of SCG before dissection occurs may yield detrimental physiological consequences rather than therapeutic benefits.

In disease pathogenesis and progression pathways, pathological states not only manifest elevated levels of pathogenic factors, but also frequently exhibit significantly upregulated expression of physiologically beneficial elements, as we found the role of TH and SCG in pre-lesion stage of AD. In models of peripheral nerve injury, the expression of distinct endogenous factors within the dorsal root ganglion, such as Adcyap1, is markedly induced following trauma, which is to attenuate nociception and foster axonal regrowth [36]. Within immunological contexts, IL-10 serves as a crucial negative feedback regulator during inflammatory and immune responses [37,38]. Its functional deficiency disrupts normally contained inflammatory processes, exacerbates tissue damage, and precipitates more severe histopathological manifestations [39–41].

We must acknowledge the main limitation in our present study. This study is conducted based on small sample size (n = 5–6 per group), which provides adequate power only to detect very large effect sizes. Thus, while our primary findings of exacerbated aortic rupture are robust, secondary molecular insights should be interpreted as exploratory.

Our research provides new insights into the pathophysiological mechanisms of AD, underscoring the critical role of the sympathetic nervous system in the early course of the disease. These findings provide a different perspective that the intact of ganglion could remaining the homeostasis of aorta. Future studies could further explore the clinical utility of TH expression levels as a biomarker and delve into the specific molecular mechanisms by which SCGx influences the progression of AD.

## Supporting information

S1 File Supplementary document. (DOCX)

S2 File Figure 1 raw images. (DOCX)

S3 File Raw data set. (ZIP)
