# Supplementary material for: Superior cervical gangliectomy exacerbates the aortic dissection in mice
Source: PLoS One. 2026 Sep 1;21(9):e0357281. doi: 10.1371/journal.pone.0357281 (PMC13533399; doi:10.1371/journal.pone.0357281)
Supplement: Supplementary file 1 [file pone.0357281.s001.docx]

**Supplemental 1 File: Supplementary document**

**Superior cervical gangliectomy exacerbates the aortic dissection** **in mice**

Zhonghui Zhu^1,2^, Hao Jin ^3^, Jie Li^1,2^, Li Yu^1,2^, Meifang Cao^3^, Tao Jiang^1^,

Yi Jin^4, *^, Baohong Jiang^1,2,3, *^

^1^Shanghai Institute of Materia Medica, Chinese Academy of Sciences, Shanghai, 201203, China;

^2^School of Pharmacy, University of Chinese Academy of Sciences, Beijing, 100049, China;

^3^School of Chinese Materia Medica, Nanjing University of Chinese Medicine, Nanjing, 210023, China;

^4^Shenzhen Institute for Drug Control (Shenzhen Testing Center of Medical Devices), Shenzhen, 518023, China

*Corresponding author jiangbh@simm.ac.cn, jinyi@szidc.org.cn

**Keywords:** Superior cervical gangliectomy, aortic dissection, aortic tensile strength, tyrosine hydroxylase

**Supplementary methods**

*Histological* *and immunohistochemical staining for SCG*

The right and left SCG were sampled and sectioned into 4 μm slices. The slices were subsequently stained with H&E as described in the main manuscript. To detect the expression of tyrosine hydroxylase (TH) or neurofilament heavy chain (NFH) on SCG, slices were incubated with antibodies against TH (1:200, cat. sc-25269, Santa Cruz) or NFH (1:1000, cat. R25134, Zenbio) at 4ºC overnight, then incubated with HRP-conjugated secondary antibody for an hour at 37 ºC. Images were captured using an Olympus BX51 microscope. Staining quantification for TH or NFH was shown as the ratio of positive area per field. Five different fields were taken for each SCG.

*Multi-segmental section of aorta*

The dissected aorta was fixed in 4% neutral-buffered formalin, and then was embedded in paraffin. The tissue was sectioned from the aortic valve, with the first segment determined when the entire aortic ring was initially identified. Subsequent sections, approximately 120 μm apart, were designated as the second segment, and so on, until two or more connected arterial rings, where the aortic arch bifurcated, were observed under the microscope, marking the last segment. Ganglions/nerve bundles around the aorta were defined as those visible within a view that included both part of the aortic structure and the ganglions/nerve bundles at 100× magnification. The number of ganglions/nerve bundles was then counted.

**Supplementary results**

**NFH was downregulated in SCG of AD mice**

To characterize the alterations of SCG in AD, histological analyses were performed first. Even no discernible morphological changes were revealed according H&E staining, preserved expression of TH alongside a marked reduction in NFH-expression in the AD group relative to Con group was observed, indicating the SCG degeneration associated with AD pathogenesis (Supplementary Fig. S1).

Supplementary Figure S1. The significant downregulation of NFH in AD mice. (A) Representative images of H&E, and the immunohistochemistry of TH and NFH on the SCG. (B) Quantification of TH and NFH in SCG (n = 8-9/group). * p < 0.05 compared with Con.

**Ganglions and nerve bundles were unchanged after SCGx**

To characterize whether SCGx influence the total ganglions and nerve bundles, multi-segmental section was performed. Results showed that the ganglions and nerve bundles kept unchanged both in AD and SCGx group (Supplementary Figure S2).

Supplementary Figure S2. The number of nerve bundles was unchanged after SCGx. Representative images of H&E stained ascending aorta sections, and quantification of ganglions/bundles in section or around aorta (n = 5/group). The ganglions and nerve bundles are indicated by *. Data are presented as mean ± SEM with individual datapoints. Group differences were assessed using Kruskal-Wallis test for scaled variables.
