# Supplementary material for: Superior cervical gangliectomy exacerbates the aortic dissection in mice
Source: PLoS One. 2026 Sep 1;21(9):e0357281. doi: 10.1371/journal.pone.0357281 (PMC13533399; doi:10.1371/journal.pone.0357281)
Supplement: Supplementary file 2 [file pone.0357281.s002.docx]

**Supplemental 2 File: Figure 1_raw_images**

**Western blot raw data**

The raw data of blots was corresponding to Fig. 1E. The protein bands were detected with an enhanced chemiluminescence (ECL) kit (abs921, Absin) and captured by Tanon 4600SF. The results were quantified by ImageJ software. Raw Figure 1 and Raw Figure 2 were used as representative images and Raw Figure 3 and Raw Figure 4 were used to enrich sample number. The total sample number was n = 5.

Raw Figure 1, corresponding to Fig. 1E up, exposure time: 1s.

Raw Figure 2, corresponding to Fig. 1E down, exposure time: 2s. The Raw Figure 1 and Raw Figure 2 were generated from one membrane according to the wave patterns we have drawn.

Raw Figure 3, not shown in figure, exposure time: 1s. It was used to quantify to enrich sample number.

Raw Figure 4, not shown in figure, exposure time: 3s. It was used to quantify to enrich sample number. The Raw Figure 3 and Raw Figure 4 were generated from one membrane according to the wave patterns we have drawn.
